# Prevalence and risk factors for diabetic retinopathy in a high-risk Chinese population

**DOI:** 10.1186/1471-2458-13-633

**Published:** 2013-07-05

**Authors:** Jiao Wang, Ru-Yi Zhang, Rong-Ping Chen, Jia Sun, Rui Yang, Xiao-Yun Ke, Hui Chen, De-Hong Cai

**Affiliations:** 1 Department of Endocrinology, Zhujiang Hospital, Southern Medical University, Guangzhou, China; 2 Department of Ophthalmology, Zhujiang Hospital, Southern Medical University, 253# industry road, Guangzhou 510282, China

**Keywords:** Finnish diabetes risk score, People with high risk for diabetes, Diabetic retinopathy, Prevalence, Risk factors

## Abstract

**Background:**

Lifestyle plays an important role in the development of diabetic retinopathy. The lifestyle in Guangzhou is different from other cities in China as the Cantonese prefer eating rice porridge, but not spicy foods. The objectives of this study were to investigate the prevalence and determinants of diabetic retinopathy in a high-risk population of Guangzhou.

**Methods:**

Subjects (619 totals) aged over 45 years old, without known diabetes were recruited from five randomly selected Guangzhou communities in 2009–2010. All participants were invited to complete the Finnish Diabetes Risk Score (FINDRISC) questionnaire. Subjects with FINDRISC score ≥ 9 were included in the study, and underwent an investigation of demographic data, a standardized physical examination, ocular fundus examination, and laboratory analyses. The minimum criterion for diagnosis of diabetic retinopathy was the presence of at least one microaneurysm.

**Results:**

Of 619 subjects, 208 eligible subjects (122 women) with FINDRISC score ≥ 9 were included in the study. The mean age was 69.2 ± 8.5 years. Diabetic retinopathy was detected in 31 subjects, and the prevalence of diabetic retinopathy in subjects with high risk for diabetes was 14.9%. In binary logistic regression analysis, risk factors associated with diabetic retinopathy were history of impaired glucose regulation [odds ratio (OR), 7.194; 95% confidence interval (CI): 1.083, 47.810], higher hemoglobin A1c (HbA1c; OR, 2.912; 95% CI: 1.009, 8.402), higher two-hour postprandial plasma glucose level (OR, 1.014; 95% CI: 1.003, 1.025), and presence of microalbuminuria (OR, 5.387; 95% CI: 1.255, 23.129).

**Conclusions:**

Diabetic retinopathy was prevalent in a high-risk Chinese population from Guangzhou. Histories of impaired glucose regulation and microalbuminuria were strong risk factors for diabetic retinopathy.

## Background

Diabetic retinopathy (DR) is a common chronic microvascular diabetic complication, and it is the leading cause of visual impairment among working adults in the Western world [1]. Apart from visual morbidity, the presence of DR may indicate microcirculatory dysfunction in other organ systems [2,3]. Therefore, investigating the prevalence of DR is important. The prevalence of diabetes mellitus (DM) and prediabetes increases with increasing age. The prevalence of prediabetes among Chinese adults aged over 45 years was dramatically higher than in those aged between 20 and 30 years [4]. However, type 2 diabetes (T2DM) is often not diagnosed until complications appear. Jia *et al.*[5] showed that the prevalence of DR in the Chinese prediabetic population was 2.5%, and Lee *et al.*[6] showed that the prevalence of diabetic retinopathy was 18.2% among recently diagnosed diabetic patients (diabetic duration ≤ 1 year) in Hong Kong, China.

Lifestyle plays an important role in the development of DR [7], and the lifestyle of Guangzhou is different from that of other cities. The Cantonese prefer eating rice porridge, but not spicy foods. The individual postprandial blood glucose level dramatically increases after eating rice porridge in a short time period, which increases the burden of pancreatic island beta (β cell function. Chaiyasit *et al.*[8] reported that people receiving capsicum had lower plasma glucose levels and higher plasma insulin levels than people in the placebo group. Capsaicin may be a useful phytochemical for attenuating obesity-related complications, such as diabetes [9]. However, the prevalence of DR has not been investigated for people with high risk for diabetes in Guangzhou.

The Finnish Diabetes Risk Score (FINDRISC) questionnaire is a fast, simple, inexpensive, noninvasive, and reliable tool to identify individuals with high risk for diabetes, and it has been widely used in different countries for identification of diabetic high-risk individuals [10-21]. In the present study, we focused on people aged over 45 years, and used the FINDRISC to identify individuals with high risk for diabetes. We then evaluated the prevalence of DR and associated risk factors.

## Methods

### Study population and design

**Figure 1:**
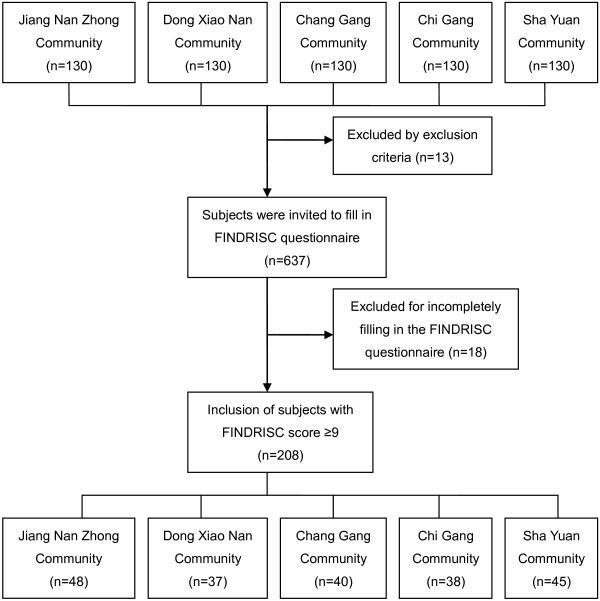
Flowchart of the samples collected.

A total of 650 subjects, aged over 45 years without known diabetes, were randomly recruited from five communities in Guangzhou between July 2009 and May 2010. Exclusion criteria were known DM, cancer, hepatic failure, renal failure, severe psychiatric disturbance, and any other systemic medical condition. Thirteen subjects were excluded from the study. All 637 subjects were invited to complete the FINDRISC questionnaire, which contained questions about age, body mass index (BMI) and waist circumference, family history of diabetes, history of antihypertensive drug treatment and impaired glucose regulation (IGR), physical activity, and daily consumption of fruits or vegetables. Eighteen subjects failed to complete the FINDRISC questionnaire, and the study response rate was 97.2% (619/637). Among 619 subjects, 208 subjects with FINDRISC score ≥ 9 were included in the study. A total of 208 included subjects received a screening for DR, a standard oral glucose tolerance test (OGTT), a physical examination (height, weight, waist circumference, hip circumference, and blood pressure), a collection of blood samples to determine the level of fasting plasma glucose (FPG), two-hour postprandial plasma glucose (2hPG) levels, glycated hemoglobin (HbA1c) levels, high-density lipoprotein cholesterol (HDL-C) concentration, low-density lipoprotein cholesterol (LDL-C) levels, total cholesterol (TC), triglycerides (TG), and a collection of first morning urine to determine the level of urinary albumin. The study protocols were conducted in accordance with the tenets of the Declaration of Helsinki and approved by the Medical Ethics Committee of ZhuJiang Hospital of Southern Medical University. All patients provided written, informed consent (Figure 1).

### Anthropometric and laboratory measurements

Weight was measured using a balance-beam scale with clothing included. Height was measured using the clinic stadiometer, with the Frankfort plane held horizontal. The BMI was calculated as weight (kg) divided by squared height (m^2^). Waist circumference was measured at the midpoint between the lowest rib margin and the iliac crest, and hip circumference was measured at the maximum circumference over the buttocks. Blood pressure was the average of three measurements obtained by a sphygmomanometer at 2-min intervals.

FPG and 2hPG were measured by the glucose oxidase method. HbA1c was measured by high-performance liquid chromatography (HLC-723G7, Tosoh, Tokyo, Japan). TC, TG, HDL-C, and LDL-C were measured by enzymatic methods. The urinary albumin level was measured by quantitative immunoturbidimetry (BN Prospect, Siemens, Berlin, Germany).

### Retinal photography and retinopathy assessment

Non-stereoscopic 45° photographs of the posterior pole were taken from both eyes of each subject obtained by a non-mydriatic fundus photographic instrument (D80, Canon, Tokyo, Japan). The fundus photographs were examined by two trained and experienced ophthalmologists. The minimum criterion for diagnosis of DR was the presence of at least one microaneurysm [22]. Retinopathy grading was based on the results of the worst eye, and the retinopathy severity score was assigned according to the International Clinical DR Disease Severity Scale [23] as follows: Grade 0, no abnormalities; Grade 1, mild non-proliferative retinopathy (microaneurysm only); Grade 2, moderate non-proliferative retinopathy (more than just microaneurysms, but less than Grade 3); Grade 3, severe non-proliferative retinopathy; Grade 4, proliferative retinopathy. Vision-threatening retinopathy (VTR) was defined as the presence of severe non-proliferative retinopathy, proliferative retinopathy, or clinically significant macular edema. All photographs were gradable, and the assessments of DR by two ophthalmologists were consistent.

### Definitions

The diagnosis of DM and IGR were previously defined based on the 1999 World Health Organization (WHO) diagnostic criteria [24]. IGR included impaired fasting glucose (6.1 mmol/L ≤ FPG < 7.0 mmol/L) as well as impaired glucose tolerance (7.8 mmol/L ≤ 2hPG < 11.1 mmol/L). Diabetes and IGR were determined based on OGTT. Prediabetes was defined as either impaired fasting glucose or impaired glucose tolerance. Hypertension was diagnosed as systolic blood pressure (SBP) ≥ 140 mm Hg and/or diastolic blood pressure (DBP) ≥ 90 mm Hg at examinations, and hypertension was also defined if the participant had a previous physician diagnosis [25]. Microalbuminuria was diagnosed as the urinary albumin concentration ≥ 30 mg/L and <300 mg/L.

### Statistical analysis

All analyses were performed using SPSS software version 13.0. Normally distributed and continuous variables were presented as mean ± standard deviation. Categorical variables were expressed as percentages, and the *χ*^*2*^ test was used for comparisons of proportions. The basic characteristics of the individuals with and without DR were compared using the Independent-Samples *t*-test for normally distributed and continuous variables. Binary logistic regression was used to assess the associations between DR and the other parameters evaluated. Odds ratios (OR) and 95% confidence intervals (95% CI) were calculated. The A value of p <0.05 was considered statistically significant (two-tailed).

## Results

### Clinical characteristics of the study population

All 619 subjects completed the FINDRISC questionnaire, 208 subjects (86 males) with FINDRISC score ≥ 9 were included in the study, and the mean age was 69.2 ± 8.5 years. Compared with subjects without DR (Table 1), subjects with DR had higher HbA1c, FPG, and 2hPG levels, higher FINDRISC scores, higher urinary albumin levels, higher ratios of microalbuminuria, and history of IGR.

**Table 1:** Clinical characteristics of the study population

	**With DR (n = 31)**	**Without DR (n = 177)**	**p value**
Age (years)	69.84 ± 7.90	69.06 ± 8.57	0.636
Gender			0.472
Male	11 (35.5%)	75 (42.4%)	
Female	20 (64.5%)	102 (57.6%)	
Body mass index (kg/m^2^)	27.31 ± 3.06	26.49 ± 3.14	0.181
Waist circumference (cm)	90.10 ± 7.03	89.66 ± 7.92	0.774
Hip circumference (cm)	100.74 ± 6.21	99.42 ± 6.55	0.296
HbA1c (%)	6.54 ± 1.38	5.59 ± 0.59	0.010
FPG (mmol/L)	6.97 ± 2.34	5.70 ± 0.94	0.005
2hPG (mmol/L)	13.71 ± 5.24	8.95 ± 3.36	<0.001
Systolic blood pressure (mm Hg)	139.23 ± 17.24	139.88 ± 19.58	0.861
Diastolic blood pressure (mm Hg)	80.39 ± 10.42	80.38 ± 10.56	0.997
Hypertension	26(83.9%)	137(77.4%)	0.420
Total cholesterol (mmol/L)	5.62 ± 1.17	5.23 ± 1.47	0.165
Triglycerides (mmol/L)	2.52 ± 2.45	2.23 ± 1.66	0.408
HDL-cholesterol (mmol/L)	1.36 ± 0.32	1.40 ± 0.33	0.545
LDL-cholesterol (mmol/L)	3.57 ± 0.83	3.47 ± 1.00	0.580
Urine albumin (mg/L)	13.9 (11.2-30.3)	11.5 (11.2-15.8)	0.022
Microalbuminuria	10(32.3%)	16(9.0%)	<0.001
Family history of diabetes mellitus	2(6.5%)	9(5.1%)	0.670
History of impaired glucose regulation	8(25.8%)	9(5.1%)	0.001
History of antihypertensive drug treatment	18(58.1%)	98(55.4%)	0.780
Physical activity (≥ 4 hours per week)	17(54.8%)	100(56.5%)	0.864
Daily consumption of fruits or vegetables	25(80.6%)	129(72.9%)	0.363
The FINDRISC score	12.06 ± 2.56	11.08 ± 2.14	0.023

*DR* diabetic retinopathy, *FINDRISC* Finnish diabetes risk score.

Among 208 subjects with a FINDRISC score ≥ 9, 55 subjects (26.4%) had DM, 84 subjects (40.4%) had IGR, and 69 subjects (33.2%) had normal glucose tolerance (NGT). There was no significant difference in prevalence of DR among five communities (p = 0.979).

### Prevalence of DR

Among 208 included subjects with a FINDRISC score ≥ 9, 27 had mild non-proliferative DR, two had moderate non-proliferative DR, one had severe non-proliferative DR, one had proliferative DR, and the total prevalence of DR was 14.9%. The prevalence of VTR was 1.4%.

As shown in Table 2, the prevalence of DR increased with increasing FINDRISC score (p = 0.041). The prevalence of patients with FINDRISC scores ≤ 10, ≤ 12, and > 12 was 8.2%, 20.3%, and 21.2%, respectively. The prevalence of DR in subjects with DM was dramatically higher than in subjects without DM (38.2% vs. 6.5%, p < 0.001). The prevalence of DR in subjects with DM was also dramatically higher than in subjects with IGR (38.2% vs. 7.1%, p < 0.001). However, there was no significant difference between subjects with IGR and subjects with NGT (7.1% vs. 5.8%, p = 0.995).

**Table 2:** Prevalence of DR in subgroups

	**Prevalence of DR**	**p value**
FINDRISC score		0.041
Score ≤ 10	8.2% (8/97)	
10 < Score ≤12	20.3% (12/59)	
Score >12	21.2% (11/52)	
Results of OGTT		<0.001
NGT	5.8% (4/69)	
IGR	7.1% (6/84)	
DM	38.2% (21/55)	
History of impaired glucose regulation		0.001
Yes	47.1% (8/17)	
No	12.0% (23/191)	
Hypertension		0.420
Yes	16.0% (26/163)	
No	11.1% (5/45)	
Microalbuminuria		0.001
Yes	38.5% (10/26)	
No	11.5% (21/182)	

*FINDRISC* Finnish diabetes risk score.

Compared with subjects without history of IGR (Table 2), subjects with history of IGR had higher prevalence of DR (47.1% vs. 12.0%, p = 0.001). Compared with subjects without microalbuminuria, subjects with microalbuminuria had higher prevalence of DR (38.5% vs. 11.5%, p = 0.001). The prevalence of DR in subjects with hypertension was slightly higher than in subjects without hypertension. However, there was no significant difference between the two groups (16.0% vs. 11.1%, p = 0.420).

### Risk factors for DR

In the present study, we carried out a full multivariable binary logistic analysis using the enter method to evaluate the risk factors for DR. The presence of DR was a dependent parameter, and age, gender, family history of DM, history of IGR, SBP, DBP, BMI, waist circumference, hip circumference, HbA1c, FPG, 2hPG, TC, TG, HDL-cholesterol, LDL-cholesterol, FINDRISC score, physical activity, daily consumption of fruits or vegetables, history of antihypertensive drug treatment, and microalbuminuria were independent parameters. As shown in Table 3, the results of binary logistic analysis showed that history of IGR (OR, 7.194; 95% CI: 1.083, 47.810) and macroalbuminuria (OR, 5.387; 95% CI: 1.255, 23.129) were highly associated with the development of DR. Every 1% increase in HbA1c increased the risk for DR by 2.912 times (OR, 2.912; 95% CI: 1.009, 8.402). Every 1 mmol/L increase in 2hPG increased the risk for DR by 1.014 times (OR, 1.014; 95% CI: 1.003, 1.025).

**Table 3:** Risk factors for DR in subjects with FINDRISC score ≥ 9

	**p value**	**Odds ratio**	**95% CI**
Age (years)	0.588	1.019	0.952-1.090
Gender	0.110	2.959	0.781-11.210
Family history of diabetes	0.541	0.492	0.051-4.784
History of impaired glucose regulation	0.041	7.194	1.083-47.810
Body mass index (kg/m^2^)	0.752	0.949	0.686-1.312
Waist circumference (cm)	0.583	0.968	0.860-1.088
Hip circumference (cm)	0.083	1.147	0.982-1.339
History of antihypertensive drug treatment	0.641	0.747	0.219-2.548
Physical activity (≥ 4 hours per week)	0.195	0.443	0.129-1.517
Daily consumption of fruits or vegetables	0.608	1.446	0.353-5.922
FINDRISC score	0.901	1.021	0.739-1.411
HbA1c (%)	0.048	2.912	1.009-8.402
Fasting plasma glucose (mmol/L)	0.126	0.972	0.937-1.008
2 h postprandial plasma glucose (mmol/L)	0.015	1.014	1.003- 1.025
Systolic blood pressure (mm Hg)	0.855	0.997	0.965-1.030
Diastolic blood pressure (mm Hg)	0.480	1.024	0.959-1.092
Total cholesterol (mmol/L)	0.189	1.486	0.823-2.683
Triglycerides (mmol/L)	0.359	0.863	0.631-1.182
HDL-cholesterol (mmol/L)	0.449	0.489	0.077-3.117
LDL-cholesterol (mmol/L)	0.661	0.830	0.362-1.907
Microalbuminuria	0.023	5.387	1.255-23.129

*FINDRISC* Finnish diabetes risk score, *HDL* high-density lipoprotein, *LDL* low-density lipoprotein.

## Discussion

Although the economy of China has made rapid progress in the past few decades, the knowledge of health care remains deficient, and not enough adequate emphasis is placed on regular physical examinations. Moreover, more than 50% of China’s population lives in rural communities, where both the economic and medical conditions are worse than in non-rural communities, and the people in rural communities often do not go to hospital to see a doctor until they have serious diseases. The prevalence of diabetes increased from 0.67% in 1980 to 9.7% in 2008 in the Chinese population, and that approximately 92.4 million of the population 20 years of age or older had diabetes [4]. However, in 60.7% of these cases, the diabetes was undiagnosed. Therefore, there is a failure to diagnose diabetes in China [4].

FINDRISC is a fast, simple, inexpensive, noninvasive, but reliable tool used to identify individuals with high risk for diabetes. It has been used in different countries for identification of diabetic high-risk individuals [10-21]. Therefore, in the present study we used the FINDRISC questionnaire to identify individuals with high risk for diabetes. No previous study has been undertaken to investigate whether the FINDRISC questionnaire could be used to identify Chinese individuals with high risk for diabetes, and what the optimal cut-off value of the FINDRISC score is in the Chinese population. Therefore, a cut-off value of the FINDRISC score ≥ 9 was used to identify subjects with high risk for diabetes, which agreed with the research findings of Lindström *et al.*[20] and Franciosi *et al.*[21]. Our studies showed that 139 subjects (66.8%) were diabetic or prediabetic in 208 subjects with FINDRISC scores ≥ 9. Therefore, our study partially confirmed that the FINDRISC questionnaire was also a reliable tool to identify individuals with high risk for diabetes in China.

Among 208 subjects with a FINDRISC score ≥ 9, the prevalence of VDR was 1.4% and the overall prevalence of DR was 14.9%. The prevalence of DR in the present study was slightly lower than in a community-based investigation from Hong Kong of 3,510 patients with recently diagnosed diabetes (18.2%) [6]. However, the prevalence of DR was dramatically higher than the prevalence rate reported in other retinal epidemiological studies in patients with newly diagnosed diabetes, such as the Shanghai Diabetic Complications Study (SHDCS, 4.9%; [5]), the Chennai Urban Rural Epidemiology Study (CURES, 5.1%; [26]), the Australian Diabetes Obesity and Lifestyle Study (AusDiab, 6.2%; [27]), and the Chungju Metabolic Disease Cohort Study (CMC study, 6.2%; [28]).

Although the FINDRISC score was not a risk factor of DR (p = 0.901), the prevalence of DR increased with an increasing FINDRISC score (*p* = 0.041). Because the sample size in this study was small, only 208 subjects were included in the study. Moreover, the distribution of the FINDRISC score was uneven, which in 87.5% (182/208) patients, was ≤ 13. We hypothesize that the small sample size and the uneven distribution of the FINDRISC score may partly explain why the FINDRISC score was not significant in logistic analysis.

Studies have shown that HbA1c was a pivotal risk factor for DR in patients with diabetes [5,22,28-32]. Consistent with previous studies, the present study showed HbA1c was an important risk factor for DR in subjects with high risk for diabetes (OR, 2.912; 95% CI: 1.009 − 8.402). Kim *et al.*[28] and Jia *et al.*[5] reported that 2hPG was an important risk factor for DR in Korean patients and Chinese patients, respectively. Consistent with the above two studies, the present study indicated that 2hPG was a risk factor for DR (OR, 1.014; 95% CI: 1.003 − 1.025).

Studies [31,33,34] have shown that microalbuminuria was one of the risk factors associated with DR. Furthermore, Chen *et al.*[33] showed that in the Chinese population a microalbuminuria threshold can predict the risk for the development of DR in T2DM. In agreement with previous studies, the present study showed that microalbuminuria was a strong risk factor for DR (OR, 5.387; 95% CI: 1.255, 23.129).

DR is a common chronic microvascular diabetic complication, and many studies had shown that higher plasma glucose level is an important risk factor for DR [5,22,28-32]. Interestingly, the present study indicates that the history of IGR is a strong risk factor for DR (OR, 7.194; 95% CI: 1.083 − 47.810). There are several reasons that may partly explain why the history of IGR is a strong risk factor for DR, and why the history of IGR is a much stronger risk factor than HbA1c or 2hPG levels. First, T2DM is often not diagnosed until complications appear. Lee *et al.*[6] reported that the prevalence of DR in patients with recently diagnosed diabetes was 18.2% in Hong Kong, China. Jia *et al.*[5] indicated that the prevalence of DR in Chinese patients with prediabetes and newly diagnosed diabetes was 2.5% and 4.9%, respectively. Second, a long-term observation study showed that approximately 70% of patients with IGR would finally progress to diabetes. Consistent with the above study, the present study showed that 52.9% (9/17) of patients with history of IGR were diabetic. Third, compared with subjects without a history of diabetes, patients with a history of diabetes had a higher ratio of microalbuminuria, higher HbA1c, and higher 2hPG levels. Further studies are needed to clarify these findings.

Some studies indicated that hypertension was a risk factor for DR in diabetes [22,27,32,35,36]. The UK Prospective Diabetes Study (UKPDS) reported that intensive control of blood pressure significantly reduced the incidence of DR [37]. However, other studies indicated that hypertension was not a risk factor for DR [5,31,38,39]. In the present study, although hypertension was not a significant risk factor for DR, the prevalence of DR in subjects with hypertension was slightly higher than in subjects without hypertension (16.0% vs. 11.1%, p = 0.420).

The present study used the FINDRISC questionnaire to identify individuals with high risk for diabetes and partly confirmed that it also was a reliable tool to identify individuals with high risk for diabetes in a Chinese population. In addition, microalbuminuria and the history of IGR were found to be risk factors for DR in the Chinese population with high risk for diabetes.

The limitations of our study should be noted. First, it was a cross-sectional investigation and the sample size was small. Second, the minimum criterion for diagnosis of DR was the presence of at least one microaneurysm. However, microaneurysm is not pathognomonic for diabetes mellitus. This may also occur in hypertension or other diseases, and hypertension and diabetic retinopathy share a number of similar morphological features, especially at their early stages [40]. Therefore, the estimated prevalence of DR may be incorrect and slightly high. Third, we used 45 photographs of the central fundus to assess and diagnose DR, rather than fundus fluorescein angiography. Thus, individuals with DR, especially at an early stage, may not be diagnosed.

## Conclusions

In summary, the FINDRISC questionnaire may also be a reliable tool to identify individuals with high risk for diabetes in China. The prevalence of DR was high in a Chinese population at high risk for diabetes. Compared with subjects with low FINDRISC scores, the prevalence of DR in subjects with high FINDRISC scores was dramatically higher. The history of IGR and the presence of microalbuminuria were strong risk factors for DR in the Chinese high-risk population. Risk factors also included higher HbA1c and 2hPG levels. As metabolic disorders are controllable, using the FINDRISC questionnaire to identify individuals with high risk for diabetes, regular screening for diabetes and DR in individuals with high FINDRISC scores, IGR, or microalbuminuria, may reduce the number of individuals who develop diabetes or DR.

## Competing interests

The authors declare that they have no competing interests.

## Authors’ contributions

JW and RYZ gave the biggest contributions to the passage; DHC gave the point about the passage; RPC, JS, and RY helped with the FINDRISC questionnaires, acquisition of data, analysis, interpretation of data, and manuscript revisions. XYK and HC analyzed the photographs. All authors read and approved the final manuscript.

## Pre-publication history

The pre-publication history for this paper can be accessed here:

http://www.biomedcentral.com/1471-2458/13/633/prepub
